# Stressors and difficulties perceived during the pandemic in the teaching activity of nursing professors

**DOI:** 10.3389/fpsyg.2022.989279

**Published:** 2022-10-13

**Authors:** Marta Losa-Iglesias, Raquel Jimenez-Fernandez, Inmaculada Corral-Liria, Elena Herraiz-Soria, Rocio Rodriguez-Vazquez, Ricardo Becerro-De-Bengoa-Vallejo

**Affiliations:** ^1^Department of Nursing and Dentistry, Rey Juan Carlos University, Móstoles, Spain; ^2^Department of Nursing, Complutense University of Madrid, Madrid, Spain

**Keywords:** anxiety, depression, resilience, academic stressors, teachers

## Abstract

**Background:**

The coronavirus 2019 (COVID-19) pandemic has prompted several changes in teaching methods in addition to the ways of learning by students.

**Objective:**

To check whether a relationship between factors, such as resilience, self-esteem, depression, anxiety, academic stressors, and a change in teaching methods and learning since the first epidemic outbreak exists.

**Materials and methods:**

This study was a cross-sectional descriptive one with a non-random sample of nursing degree teachers who did or did not participate in clinical activities but had been teaching online since the start of the pandemic. Data were collected with online questionnaires validated for self-completion with Google Forms.

**Results:**

Regarding the analysis of the descriptive data of each scale, we can verify that data indicate very high levels of resilience and self-esteem in the normal range with minimal levels of depression, moderate anxiety, and finally not worrying about sources of stress in teachers. Also, negative correlations were found between the 10 Connor–Davidson Resilience Scale, Beck Depression Inventory (BDI 2), Beck Anxiety Inventory (BAI), and Scale of Sources of Stress in Teachers with a statistical significance of *p* < 0.001. The Rosenberg Self-Esteem Scale also showed negative correlations with the three previously mentioned scales with a statistical significance of *p* < 0.001. Finally, positive correlations between the Beck (BDI 2), Beck (BAI), and Sources of Stress in Teachers scales and between the Rosenberg Self-Esteem scale and the 10 CD RISC scale were found (*p* < 0.001).

**Discussion:**

Our study shows that nursing degree teachers combine teaching with activities and presented moderate levels of anxiety, depression, and tolerance to academic stressors and were able to maintain optimal levels of self-esteem and resilience, indicating that these two factors act as protectors against these stressors.

**Tweetable abstract:**

Nursing teachers presented moderate levels of anxiety, depression, and academic stressors due to optimal levels of self-esteem and resilience.

## Background

The coronavirus 2019 (COVID-19) pandemic has caused profound social changes. In the university, especially during the first wave, a rethinking of teaching methods and ways to receive feedback from students has occurred (Barry and Kanematsu, 2020; Pokhrel and Chhetri, 2021).

Due to the ongoing pandemic with different pandemic spikes during the academic year 2020–2021, universities in general throughout the world and in Spain, in particular, adopted new teaching models for teaching health sciences, which also includes nursing. During the pandemic, some universities established a learning online model and exclusively physical presence for clinical and preclinical practices, because the classrooms were not prepared to maintain social distancing measures (Leigh et al., 2020).

The sudden change in the teaching model combined with pandemic fatigue in our field and in the university is reflected throughout the university community as a sense of abandonment, sadness, lack of motivation, and job performance below the average of the years before the pandemic (Torda et al., 2020; Day et al., 2021; Santos et al., 2021). The group of teachers with less experience in new technologies is the one that suffered and is suffering the most with this change (Rasheed et al., 2020). Also in healthcare professions, such as nursing, in which the face-to-face component has been established as being a very important aspect for which to acquire skills and competencies, teachers have been subject to greater pressure to reconcile non-face-to-face teaching with the decrease in teaching quality (Costa et al., 2020).

Preliminary studies have shown the urgent need to develop preventive interventions and strategies to address the mental health of university professors (Brooks et al., 2020; Besser et al., 2022) as teachers show psychological stress linked to the symptom of somatization, depression, anxiety, and/or catastrophic thoughts in addition to academic, health, and lifestyle concerns directly caused by the pandemic (Wang et al., 2020; Amaral-Prado et al., 2021). In a study carried out with nursing professors, up to 16 pandemic-related psychological consequences were described with depression, decreased concentration, and apathy as the most important ones (Sepahvand and Taghipour, 2020).

## Objective

For these reasons, the main objective of this research was to assess whether a relationship between factors, such as resilience, self-esteem, depression, anxiety, academic stressors, and changes in teaching and learning methods exists and to check whether the pandemic is having an impact on teachers of the nursing degree who have taught online continuously since the first epidemic outbreak. Also, it shows if the nursing professors with clinical activity in the pandemic had different levels from professors without clinical activity.

## Materials and methods

### Design and setting

A cross-sectional descriptive study with a non-random sampling of nursing degree teachers who participated or did not participate in clinical activities and who had been teaching online since the start of the pandemic was conducted at the Rey Juan Carlos University of Madrid-Spain with a total of 55 professors teaching nursing degrees. The data were collected with online questionnaires validated for self-completion with Google Forms.^1^

The Google Forms included an informed consent sheet, sociodemographic variables (age, sex, marital status, weight, height, employment status within the Universidad Rey Juan Carlos, children and dependents in their charge, whether they had COVID-19 during the past course), and the selected validated questionnaires: (1) Rosenberg Self-Esteem Scale or RSE (Rosenberg, 1989; Atienza et al., 2000); (2) Connor–Davidson Risk Resilience Scale or CD-RISC (Connor and Davidson, 2003; Notario-Pacheco et al., 2011); (3) Beck Anxiety Inventory or BAI (Beck et al., 1988); (4) Inventario de Beck Depression Inventory or BDI, BDI-II (Beck et al., 1996); and (5) Escala de Fuentes de Estrés en Profesores or EFEP “Scale of Sources of Stress in Teachers” (Nogareda, 2000). Finally, an open question asking for any kind of academic stressor not shown on the scales but important for informants was given.

### Sample

Participants were drawn from the Professor’s staff of the Degree of Nursing at Universidad xxx xxxx, xxx. The study took place between October 1, 2021 and November 29, 2021.

The sample size was calculated with software from Unidad de Epidemiología Clínica y Bioestadística, Complexo Hospitalario Universitario de A Coruña, Universidade A Coruña.^2^ From a sample of 55 individuals with an α error of 0.05, a confidence level (CI) of 95%, and heterogeneity of 50%, the required number of final participants was calculated to be 49. Finally, the final sample consisted of 53 staff nurses and nurses’ professors who had worked for a minimum of 3 years in the Universidad xxx xxxx, xxx. The inclusion criteria consisted of two parameters: (1) nursing professors and nurses from the Universidad xxx xxxx, xxx of the Nursing Degree with a minimum of 3 years of teaching at that University and (2) adequate knowledge of Spanish in both oral and written levels. The exclusion criterion was inadequate completion of the questionnaires.

### Assessment scales

#### Rosenberg self-esteem scale

This questionnaire consists of 10 questions, scored from 1 to 4 points (4 = strongly agree, 3 = agree, 2 = disagree, 1 = strongly disagree); five statements have a positive direction, and five have a negative direction. The authors of the questionnaire did set limits for this scale, but a range of scores between 20 and 30 points is usually considered a normal range. If the score is greater than the normal range, such a result would indicate high self-esteem, whereas if the result is less than normal, low self-esteem is indicated. The RSE shows strong convergent validity for men and women from different ethnic groups (Robins et al., 2001). The scale has high reliability, with test-retest correlations in the range of 0.82–0.88 (Rosenberg, 1989) and 0.87 for the Spanish population (Atienza et al., 2000).

#### 10 CD-risk, Connor–Davidson risk resilience scale

Resilience was evaluated using the short version of the Connor–Davidson Risk Resilience Scale (CD-RISC) which was validated in Spanish by Notario-Pacheco et al. (2011). The scale consists of 10 items (those numbered 1, 4, 6.7, 8, 11, 14, 16, 17, 19) from the original scale designed by Connor and Davidson (2003).

Using this scale, participants were asked to answer to what extent they agree with each of the sentences presented to them (for example, item 1: “I am able to adapt to changes.” The response format is a five-point Likert-type scale from 0 to 4.

The final score is the sum of all the responses obtained for each item (range 0–40), and the highest scores indicate the highest level of resilience.

The reliability of the 10-item CD-RISC is defined by a Cronbach’s alpha of 0.85, and the weights in factor analysis are within the range of 0.48–0.76 (Notario-Pacheco et al., 2014).

#### Beck anxiety inventory

The Beck Anxiety Inventory (BAI) questionnaire contains a list of 21 symptoms indicating anxiety with a 4-point Likert scale ranging from not at all to severe and the degree to which each symptom affected them during the last week. The values of each element were added up, and a total score ranging from 0 to 63 points was obtained. A total score from 0 to 7 was interpreted as a minimum level of anxiety, from 8 to 15 as mild, from 16 to 25 as moderate, and from 26 to 63 as severe (Beck et al., 1993). Also, in the adapted version for the Spanish population, the instrument showed high internal consistency with a Cronbach’s alpha coefficient of 0.92 and test-retest reliability of 0.75. The BAI has a high internal consistency (Cronbach’s alpha from 0.90 to 0.94). The correlation of the items with the total score ranges between 0.30 and 0.71. The test-retest reliability after 1 week ranged from 0.67 to 0.93, and after 7 weeks, the reliability was 0.62 (Comeche et al., 1995).

#### Beck depression inventory (BDI, BDI-II)

The Beck Depression Inventory (BDI) is a questionnaire with a group of 21 items, all questions used a Likert scale for answers. The internal consistency measure was alpha = 0.78. Sample items (sadness, for example) included responses, such as “I feel sad most of the time” or “I don’t feel sad.” The original BDI-II manual (Beck et al., 1996, p. 11) proposed the following cut-off estimates and corresponding depression grades: (1) 0–13 indicates minimal depression, (2) 14–19 mild depression, (3) 20–28 moderate depression, and (4) 29–63 severe depression. The Spanish adaptation of Sanz and Vázquez (1998) assumes the cut-off scores designed by Beck et al. (1996), and the reliability of the instrument is high both in terms of internal consistency (Cronbach’s alpha coefficient = 0.83) and temporal stability (test-retest correlations ranged between 0.60 and 0.72 for three different subgroups of the total sample).

#### Escala de Fuentes de Estrés en Profesores, “Scale of sources of stress in teachers”

To detect sources of stress, the Scale of Stress Sources in Teachers (EFEP) was a questionnaire created and validated by the National Institute of Safety and Hygiene at Work (2000) in Spain with which sources of stress are detected and rated on intensity. The questionnaire contains 56 items, answered using a Likert scale from “a lot” to “nothing.” The total score on the stress scale ranges from a minimum score of 56 to a maximum of 280. Three levels of stress are established on this scale: (1) green level or not worrying; score between 56 and 140; (2) yellow level or worrying; score between 141 and 196; and (3) red level or severe; score above 196. It is also convenient that the data are analyzed item by item to establish a ranking of the items that generate more academic stress (Nogareda, 2000).

In addition, an open question was included so that any individual who considered that a stressor was missing from the list of the questionnaire could let us know.

### Ethical considerations

All participants marked the point of conformity of the informed consent before completing the questionnaire provided in the Google link. The study was approved by the Ethics Committee of the Universidad Rey Juan Carlos (2910202121221 number).

### Data analysis

All variables were examined for normal distribution using the Kolmogorov–Smirnov test, and data were considered normally distributed if *p* > 0.05.

An analysis of quantitative variables was performed using means and deviations, and for categorical variables, counts and percentages were used. Spearman’s correlation was performed to measure the strength of the association between quantitative variables. Pearson’s correlation and Mann–Whitney *U* tests were performed to verify associations between variables. All statistics were considered statistically significant at *p* < 0.05 (SPSS for Windows, version 20.0; SPSS Inc., Chicago, IL, USA). The open question was analyzed using the Nvivo 8 program to illustrate the answers, findings, and interpretations on a digital mental map. An analysis of the content of the answers was carried out following a series of steps, including the selection of keywords, which were grouped following a morphological criterion to later form categories and subcategories, and finally, the word cloud was eliminated. These clouds were interpreted following a spiral arrangement since the most repeated responses were displayed as larger text and in the center of the cloud.

## Results

All variables showed a non-normal distribution (*p* < 0.05) for age, weight, height, and body mass index (BMI) *p* > 0.05) as shown in Table 1.

**TABLE 1:** Descriptive data of the participant’s total population.

Descriptive data	Total group	Male	Female	*P-value* *
	Mean ± SD (95% CI) *N* = 53	Mean ± SD (95% CI) *n* = 16	Mean ± SD (95% CI) *n* = 37	
Age (years)	45.43 ± 8.50 (43.08–47.77)	39.35 ± 9.85 (35.37–43.33)	44.64 ± 9.16 (41.59–47.70)	0.155
Weight (kg)	73.11 ± 20.66 (67.41–78.80)	69.63 ± 15.48 (63.38–75.89)	67.48 ± 21.03 (60.47–74.50)	<0.001
Height (m)	1.65 ± 4.99 (1.60–1.70)	1.66 ± 0.10 (1.62–1.70)	1.60 ± 19.55 (1.54–1.67)	<0.001
BMI (Kg/m^2^)	25.03 ± 4.37 (23.83–26.24)	25.08 ± 4.62 (23.22–26.95)	23.92 ± 4.20 (22.52–25.32)	<0.001

BMI, body mass index; Kg, kilograms; M, meters; SD, standard deviation; CI, confidence interval. *Independent *t*-student was applied. In all analyses, *p* < 0.05 (with a 95% confidence interval) was considered statistically significant.

Regarding the analysis of the descriptive data of each scale, it was verified that very high levels of resilience (31.11 ± 5.94), self-esteem in the normal range (29.88 ± 4.68), minimal levels of depression (10.69 ± 9.27), moderate anxiety (13.20 ± 14.20), and finally, in the green range, not worrying based on the Sources of Stress in Teachers scale (136.50 ± 56.97) as shown in Table 2.

**TABLE 2:** Descriptive data based on different assessment scales.

Scale	Mean (SD) (CI 95%)	Median (CI 95%)
10 CD RISC	31.11 ± 5.94 (29.47–32.75)	32.00 (29.89–35.00)
Beck (BDI 2)	10.69 ± 9.27 (8.14–13.25)	8.00 (5.00–13.20)
Beck (BAI)	13.20 ± 14.20 (9.29–17.12)	8.00 (5.00–14.00)
Rosemberg self-steem	29.88 ± 4.68 (28.59–31.17)	31.00 (29.00–32.00)
Scale of sources of stress in teachers	136.50 ± 56.97 (120.80–152.21)	143.00 (108.79–162.51)

SD, standard deviation; CI, confidence interval.

In the top 10 ranking of academic stressors, the one that caused the most stress was “How much my academic activity is valued by others” followed by “Receiving incompatible or opposite instructions,” “Teaching classes in a language that is not my mother tongue,” “I do not define my responsibilities,” “Witnessing aggression among students,” “Lack of information about how I should do my job,” “Lack of opportunities for promotion,” “Inconsideration by students,” “Pressures within the center to obtain certain results,” and “Poorly defined work schemes.”

The analysis of correlations between the different scales shows negative correlations between the 10 CD RISC scale and the Beck (BDI 2), Beck (BAI), and Sources of Stress in Teachers scales with a statistical significance of *p* < 0.001 in addition to the Rosenberg Self-Esteem Scale, which also shows negative correlations with the Beck (BDI 2), Beck (BAI), and Sources of Stress in Teachers scales with a statistical significance of *p* < 0.001. Finally, positive correlations between the Beck (BDI 2), Beck (BAI), and Sources of Stress in Teachers scales and between the Rosenberg Self-Esteem and the 10 CD RISC scales with a statistical significance of I < 0.001 as shown in Table 3.

**TABLE 3:** Correlation and differences between scales.

Scale	10 CD RISC	Beck (BDI 2)	Beck (BAI)	Rosemberg self-steem	Sources of stress in teachers
10 CD RISC	1				
Beck (BDI 2)	–0.532 (<0.001)	1			
Beck (BAI)	–0.581 (<0.001)	0.802 (<0.001)	1		
Rosemberg self-steem	0.645 (<0.001)	–0.785 (<0.001)	–0.675 (<0.001)	1	
Scale of sources of stress in teachers	–0.407 (<0.001)	0.432 (<0.001)	0.285 (0.038)	–0.431 (0.001)	1

rho (*p*-value) Spearman’s correlation coefficient; statistical significance for a *P* < 0.05, with a 95% confidence interval (CI).

### Differences between socio-demographic variables in the four questionnaires

Analyzing the differences between sexes, significant data from the Beck questionnaires, BDI 2 (*p* = 0.003), and BAI (*p* = 0.029) were found (Table 4).

**TABLE 4:** Differences in sex.

Scale	Female (*n* = 37)		Male (*n* = 16)		*P-value*
	Mean (SD) (CI 95%)	Median (CI 95%)	Mean (SD) (CI 95%)	Median (CI 95%)	
10 CD RISC	31.78 ± 5.66 (29.73–33.82)	32.00 (29.47–32.75)	30.09 ± 6.35 (27.20–32.98)	31.00 (26.24–35.00)	0.361
Beck (BDI 2)	9.25 ± 8.25 (6.27–12.22)	7.00 (3.00–15.00)	12.90 ± 10.48 (6.13–17.67)	8.00 (5.56–19.87)	0.003
Beck (BAI)	11.37 ± 13.08 (6.65–16.09)	6.50 (2.00–13.00)	16.00 ± 15.66 (8.86–23.13)	10.00 (5.00–18.75)	0.029
Rosemberg self-steem	30.06 ± 4.70 (28.36–31.75)	31.00 (28.99–32.00)	29.61 ± 4.74 (27.45–31.78)	31.00 (27.56–32.43)	0.108
Scale of sources of stress in teachers	128.09 ± 57.84 (107.23–148.25)	110.50 (85.99–159.00)	149.33 ± 54.45 (124.54–174.12)	167.00 (120.72–180.19)	0.337

SD, standard deviation; CI, confidence interval; *p*-value from Mann–Whitney *U*-test; Statistical significance for a *p* < 0.05, with a 95% confidence interval (CI).

No significant differences between professors with and without clinical activity, professors who contracted COVID-19 (or not), and marital status were found (Table 5).

**TABLE 5:** Differences between professors with and without clinical activity and differences in those with/without coronavirus 2019 (COVID-19).

Scale	Professor without clinical activity (*n* = 32)		Professor with clinical activity (21)		*P-value*
	Mean (SD) (CI 95%)	Median (CI 95%)	Mean (SD) (CI 95%)	Median (CI 95%)	
10 CD RISC	31.78 ± 5.66 (29.73–33.82)	32.00 (30.00–35.00)	30.09 ± 6.35 (27.20–32.98)	31.00 (26.24 –35.00)	0.343
Beck (BDI 2)	9.25 ± 8.25 (6.27–12.22)	7.00 (3.00–15.00)	12.90 ± 10.48 (6.13–17.67)	8.00 (5.56 –19.87)	0.142
Beck (BAI)	11.37 ± 13.08 (6.65 –16.09)	6.50 (2.00–13.00)	16.00 ± 15.66 (8.86–23.13)	10.00 (5.00–18.75)	0.201
Rosemberg self-steem	30.06 ± 4.70 (28.36–31.75)	31.00 (28.99–32.00)	29.61 ± 4.74 (27.45–31.78)	31.00 (27.56–32.43)	0.776
Scale of sources of stress in teachers	128.09 ± 57.84 (107.23 –148.25)	110.50 (85.99–159.00)	149.33 ± 54.45 (124.54 –174.12)	167.00 (120.72–180.19)	0.138
**Scale**	**COVID-19 (*n* = 35)**		**NO COVID-19 (*n* = 18)**		
	**Mean (SD)** **(CI 95%)**	**Median** **(CI 95%)**	**Mean (SD)** **(CI 95%)**	**Median** **(CI 95%)**	***P-value***
10 CD RISC	32.05 ± 6.76 (28.69–35.41)	32.00 (28.79–38.00)	30.62 ± 5.52 (28.73–32.52)	32.00 (29.00–34.75)	0.258
Beck (BDI 2)	7.97 ± 8.34 (3.79–12.09)	4.00 (2.39–14.01)	12.11 ± 9.52 (8.84–15.38)	10.00 (7.00–15.75)	0.090
Beck (BAI)	12.83 ± 15.84 (4.95–20.71)	5.50 (2.00–16.01)	13.40 ± 13.51 (8.75–18.04)	11.00 (5.00–16.00)	0.540
Rosenberg self-esteem	30.50 ± 5.46 (27.78–33.21)	32.00 (29.39–34.60)	29.57 ± 4.27 (26.10–31.03)	30.00 (28.00–31.00)	0.192
Scale of sources of stress in teachers	140.22 ± 70.20 (105.31–175.13)	138.00 (79.17–185.00)	134.60 ± 49.88 (117.46–151.73)	143.00 (109.00–159.00)	0.873

SD, standard deviation; CI, confidence interval; *p*-value from Mann–Whitney *U* test; statistical significance for a *p* < 0.05 with a 95% confidence interval (CI).

#### Analysis of the contents of the open-ended question

**FIGURE 1:**
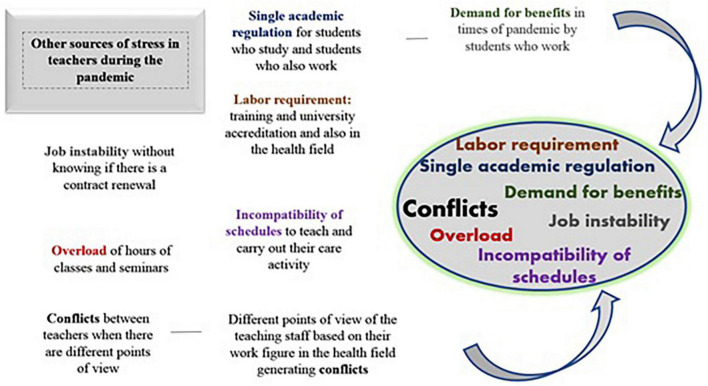
Digital mind map derived from the open-ended study question and used to illustrate simulation-based answers, feelings, and experiences.

By analyzing the content of the teachers’ answers about other sources of stress not mentioned in the questionnaires and using the Nvivo 8™ program, some common responses, emotions, and experiences were revealed. The main and most repeated topic was the possible conflicts between teachers due to discrepancies in teaching methods and ways to address these conflicts. Teachers also expressed great concern about job stability during this pandemic period and how their daily work has been overloaded with new academic responsibilities (Figure 1).

## Discussion

The main objective of this research was to determine the relationship between factors, such as resilience, self-esteem, depression, anxiety, and academic stressors on nursing teachers and the impact of these factors on these teachers. In light of the results, it was verified that the teachers were able to withstand the emotional demands and work overloads with a new and different method of teaching due to optimal levels of self-esteem and resilience. We also found that men had higher levels of depression and anxiety than women in the study with no differences between the sexes with respect to the other analyzed variables.

Regarding our main results, maintaining adequate levels of self-esteem and resilience was manifested as essential to avoid becoming depressed and anxious; these same results were verified in previous studies that demonstrate that people with high levels of resilience and self-esteem possess better problem-solving coping strategies than those showing lower levels (Morales-Rodríguez, 2021; Özdemir and Adıgüzel, 2021; Janzarik et al., 2022).

In a recent systematic review (Ozamiz-Etxebarria et al., 2021), the results suggest that teachers are experiencing adverse psychological symptoms during the COVID-19 pandemic and that anxiety levels vary between different countries. Therefore, based on our results, educational organizations should consider and encourage these teachers to maintain optimal levels of self-esteem and resilience. The negative effects of this prolonged stress and anxiety have already been reported as post-traumatic stress disorder among teachers in other studies (Kukreti et al., 2021).

Our results also point to gender differences with respect to levels of anxiety and depression with men reporting the highest levels of anxiety and depression in this study. Our results are different from other studies reviewed in the scientific literature that show that female teachers have higher levels of stress and anxiety than male teachers. In these other studies involving primary and secondary teachers, it was shown that women obtained high stress, anxiety, and depression scores (Jakubowski and Sitko-Dominik, 2021; Ozamiz-Etxebarria et al., 2021; Santamaría et al., 2021). In studies involving hospital nurses (without academic activity), female nurses were found to have higher levels of anxiety and depression (Liu et al., 2021). The explanation for the differences between those studies and our results is the different makeup of university professors in addition to depression and anxiety levels that were also well below those shown by the other studies. Specifically, our sample obtained optimal scores for resilience and self-esteem in such a way that comparing our study to other studies is more complicated.

Regarding academic stressors, the professors in our study reported moderate levels for the stressors that have the most negative impact on work, such as those related to the value given to academic work by others in addition to contradictory orders or excessive demands of work and pressure to obtain results without considering conflicts and the possibility of witnessing violence in the classroom. These data are consistent with both sets of data obtained from the questionnaire and those obtained from the open question. Our results coincide with a recent study in which pressure on academic efficacy is a very stressful factor for faculty members (Han et al., 2021). In another article, it was clearly stated that the lack of academic recognition together with overload, conflicts, and salaries below expectations are sources of academic stress and are analogous to results collected by the survey and the open question of our study (Ezenkiri et al., 2021). Balancing the workload together with clear orders from bosses can provide a good strategy for reducing academic stress as shown in the article by Lee et al. (2022) in conjunction with our results.

Some limitations of this study should be discussed. First, it is a convenient sampling technique that may produce partial outcomes so that findings are not universal. Another limitation is the sample that consists of only university nursing degree teachers, so the applicability of our results should be limited to this type of faculty. Therefore, increasing the sample by adding professors from other health sciences degrees together with random sampling could consolidate these results.

## Conclusion

The pandemic has caused a sudden divergence in teaching and learning methods in universities around the world, so understanding the impact that these changes have had and are producing is essential for educational organizations. Our study shows that nursing degree teachers, even though many of them combine teaching and care activities, reported moderate levels of anxiety, depression, and tolerance to academic stressors largely due to optimal levels of self-esteem and resilience, a result indicating that showing these two factors act as protectors for them.

### Relevance for clinical practice

Nursing teachers combine teaching with activities and presented moderate levels of anxiety, depression, and tolerance to academic stressors and were able to maintain optimal levels of self-esteem and resilience, indicating that these two factors act as protectors against these stressors. Balancing the workload together with clear orders from bosses can provide a good strategy for reducing academic stress.

## Data availability statement

The data supporting the conclusions of this article will be made available by the authors upon reasonable request.

## Ethics statement

The studies involving human participants were reviewed and approved by the Ethics Committee of the Universidad Rey Juan Carlos (2910202121221 number). The patients/participants provided their written informed consent to participate in this study.

## Author contributions

ML-I and RJ-F: substantial contributions to conception and design, acquisition of data, and analysis and interpretation of data; drafting the article or revising it critically for important intellectual content; and final approval of the version to be published. IC-L, EH-S, and RR-V: substantial contributions to conception and interpretation of data and final approval of the version to be published. RB: critical revision of the manuscript for important intellectual content and final approval of the version to be published. All authors contributed to the article and approved the submitted version.

## Footnotes

1
https://forms.gle/uNYrWncYUeUDXKG5A

2
www.fisterra.com
